# The Story of the Insane from Year to Year

**Published:** 1898-10-15

**Authors:** 


### THE STORY OF THE INSANE FROM YEAR TO YEAR.

Eleventh Series.

### THE PRESTWICH ASYLUM, MANCHESTER.

On January 1st, 1897, this asylum contained the enormous number of 2,599 patients, and on the last day of the year even this number had been added to, for we notice it then had on the books the names of 2,639 lunatics. During the year there were 428 admissions, 87 readmissions, 228 discharged recovered, 68 discharged relieved, and 179 deaths. The percentage of recoveries on admissions was 45·42. This is a high proportion, and, so far as it goes, may be taken to demonstrate that a very large mass of patients is not of itself any barrier to a high recovery rate. The deaths, calculated on the average number resident, were only 6·44 per cent., which is about as much below the usual average as the recovery rate is above it. Of the deaths 84 were caused by various cerebral and spinal diseases, 22 by consumption, and 28 by pneumonia. We find that in 166 of the year's admissions the insanity was due to various moral causes, including domestic troubles, love, religion, and fright, and so on; while among the physical causes intemperance in drink accounts for 124, heredity for 110, puerperal state for 26; and in 76 cases the insanity was of unknown origin. It will be seen that in 234 of the 515 admissions preventable causes were at work, and, high as this proportion is, it would still be higher if we could learn the causes in the 76 classed as of unknown origin, and of the 133 in which the insanity is said to have been caused by previous attacks. We may well ask once again how much longer English people are to be allowed to drive themselves mad with drink and to propagate a race of lunatics? Mr. Ley ably expresses what almost every medical superintendent has at one time or other expressed before, that patients of the criminal class should not be admitted into our county asylums. "As a rule," says Mr. Ley, "these persons have all their bad qualities intensified by their insanity, and, being perfectly well aware of the privilege their insanity affords, are actively antagonistic to the maintenance of the necessary order and discipline of a large establishment." Another point is that the ordinary patients should not be compelled to associate with criminals. If the last Lunacy Act had dealt with this question, it might have been pardoned for the insertion of a good deal better left out.

Three of the attendants were granted pensions of £56, £52, and £38 respectively, and we congratulate Samuel Maginnis, Frederick Bond, and Joseph Fletcher on having obtained these allowances. The weekly cost per head is 8s. 4⅝d.

